# The 15-year national trends of genital cancer incidence among Iranian men and women; 2005–2020

**DOI:** 10.1186/s12889-023-15417-0

**Published:** 2023-03-15

**Authors:** Gita Shafiee, Amir-hossein Mousavian, Ali Sheidaei, Mehdi Ebrahimi, Fatemeh Khatami, Kimiya Gohari, Mohammad Jabbari, Ali Ghanbari-Motlagh, Afshin Ostovar, Seyed Mohammad Kazem Aghamir, Ramin Heshmat

**Affiliations:** 1 Chronic Diseases Research Center, Endocrinology and Metabolism Population Sciences Institute, Tehran University of Medical Sciences, No. 111, 19th St., North Kargar Ave., Tehran, Iran; 2 Department of Epidemiology and Biostatics, School of Public Health, Tehran University of Medical Sciences, Tehran, Iran; 3 Department of Internal Medicine, Faculty of Medicine, Sina Hospital, Tehran University of Medical Sciences, Tehran, Iran; 4 Urology Research Center, Sina Hospital, Tehran University of Medical Sciences, Hasan Abad Sq, Tehran, Iran; 5 Department of Biostatistics, Faculty of Medicine Sciences, Tarbiat Modares University, Tehran, Iran; 6 Department of Radiotherapy, School of Medicine, Shahid Beheshti University of Medical Sciences, Tehran, Iran; 7 Osteoporosis Research Center, Endocrinology and Metabolism Clinical Sciences Institute, Tehran University of Medical Sciences, Tehran, Iran

**Keywords:** Genital cancer, Prostate cancer, Cervical cancer, Testicular cancer, Ovarian cancer

## Abstract

**Background:**

Cancer is a major health problem and cause of mortality worldwide. Despite the prevalence of other cancers in males and females, genital cancers are especially important because of their psychological effects on individuals. Currently, cervical cancer, corpus uteri neoplasm, and ovarian cancer are the most common gynecological cancers in Iran. Prostate cancer has increased in Iranian men in the last decade. Therefore, this study aimed to investigate the 15-year national trend in the incidence of genital cancers in the Iranian population.

**Methods:**

In this study, we used Iranian cancer registration data collected by the Ministry of Health and Medical Education, demographic information from the reports of the Statistics Center of Iran, STEPs (STEPwise approach to non-communicable diseases risk factor surveillance), and Caspian (childhood and adolescence surveillance and prevention of adult non-communicable disease). A list of potential auxiliary variables and secondary variables at all levels of the province-age-sex were evaluated during the years. We used mixed-effects Poisson regression to model the data and calculate the incidence of each cancer.

**Results:**

Our results show an enhancement in the outbreak of all types of male cancers, but the most important are prostate (11.46 in 2005 to 25.67 in 2020 per 100,000 males) and testicular cancers (2.39 in 2005 to 5.05 per 100,000 males). As for female cancers, there has been an increase in ovarian and corpus uteri neoplasm incidence with 6.69 and 4.14 incidences per 100,000 females in 2020, making them the most occurring female genital neoplasms. While the occurrence of cervical cancer has decreased over the years (4.65 in 2005 to 3.24 in 2020). In general, the incidence of genital cancers in men and women has amplified in the last 15 years.

**Conclusions:**

Our study examined the trend of change for each malignant genital neoplasm for 15 years in Iranian men and women in each province. Considering the growing trend of the elderly population in Iran, patient awareness and early screening are essential in reducing mortality and costs imposed on patients and the health care system.

**Supplementary Information:**

The online version contains supplementary material available at 10.1186/s12889-023-15417-0.

## Background

Cancer is a major worldwide healthcare concern and the cause of many deaths [1]. Despite medical breakthroughs and technological advancements in the prevention and treatment of cancer, the prevalence of people diagnosed with cancer has been on an upward trend in all countries [1]. In 2020, about 19.3 million new cancer cases and over 10.0 million cancer deaths occurred in the world, using the GLOBOCAN report [2]. According to a study by The Global Burden of Diseases (GBD), number of cancers detected and total number of deaths due to cancer has risen by 24.6% and 20.9% from 2010 to 2019 respectively [3]. With the vast increase in incidences, cancer has become one of Iran’s leading causes of death [4].

Female breast cancer (11.7%), lung (11.4%), and colorectal (10.0%) cancers are the most common cancers in the world [2]. In addition to the common cancers in both sexes, genital cancers are important because of their psychological effects due to loss of genital parts or infertility regardless of gender [2].

The American Cancer Society estimated that approximately 1.9 million new cases of cancer are diagnosed in 2021. Of these, a total 376,970 people have genital cancers, which consists of 260,210 and 116,760 newly diagnosed cases in men and women, respectively [5]. In more detail, cervical cancer accounts for the most common type of genital cancers among women worldwide and according to the global classification in 2020, this cancer with an incidence of 3.1% (604,127 of new cases), ranks as the eighth most common cancer in women [2, 6]. In Iran, the incidence rate of genital cancers has increased from 2.5 to 12.3 per 100,000 women from 1990 to 2016, while Cervical cancer, corpus uteri neoplasm and cancer of ovary are the most common gynecological cancers in Iran [7, 8].

Prostate cancer is the second most dominant type of cancer and the fifth cause of cancer mortality among men in the world [2]. Since the population of men over 65 years is growing, the number of subjects diagnosed with prostate cancer will increase in the near future [9]. In Iran, the incidence of prostate cancer has increased in the past decade and is currently higher than other Asian countries [4]. Based on the findings from 2011 to 2015 in Iran, the mean age of genital cancers in men was greater than women, with peak incidence at the age of 70–80 years in men and 50–60 years in women [7, 10].

In general, according to global and Iran statistics on the growing prevalence of genital cancers and given the fact that the incidence of this types of cancer depends on numerous factors such as age, sex, geographical location, lifestyle and race [11–13], a specific and comprehensive study of these malignancies in both genders, is not available in Iran yet. Moreover, there has been no comparison or evaluation of these types of neoplasms. Therefore, considering the importance of this type of study in preventing and reducing the economic costs of health care and improving quality of life, we investigated this issue.

## Methods

### Data sources

We used data from the Iranian population-based cancer registry, gathered by the Ministry of Health and Medical Education from all medical facilities. Individual data were available for 2008 to 2010, 2014, and 2015. The information in data includes ICD10 codes for neoplasm type, age, sex, and the province of residence. There were a few missing values for each variable and the proportion of missing were less than 5%, therefore we imputed them using the multiple imputation bootstrapping-based algorithm by Amelia package in R software [14].

Several scenarios for age groups definition were considered including the length of groups, optimal cut points, the minimum valid age, and the way of definition for the last group. For starting age, we relied on the global burden of diseases (GBD) study and set it at 15 years old [3]. Conducting fivefold cross-validation revealed the 10-years length age group has a lower mean square error than alternative approaches especially 5-years length age groups [15]. In addition, the selection of more than 75 years old as the latest age group showed better model performance comparing with more than 85 years. Therefore, the age groups in this study start from 15 and the groups include 10 years until the last one that is more than 75 years (15–24, 25–34, etc., and 75 + years old).

The population data were extracted from reports of the Statistical Center of Iran (SCI) for population and housing census 2001, 2006, 2011, and 2016 [16]. The data set was formed according to age and sex groups for each sub-national division. In order to estimate the population for the years between two consecutive censuses, the growth formula for the population was used [17]. The growth rate was calculated and applied separately for each subgroup of the dataset. For the years between 2017 and 2020, the growth rate of the period 2011 to 2016 was used.

The connection between cancer registry information and covariates was not possible in individual level. Therefore, we select an ecological approach rather than a cross sectional study. In this manner subjects are groups of individuals who were living in a same province as geography characteristic and were in the same sex-age group. This approach enabled us to use covariates from other sources of information.

We prepared a list of potential covariates for modeling section according to relevancy and availability of data. There were two national survey study that are conducting regularly in Iran health system. Both surveys have representative sample and follow the World Health Organization (WHO) guidelines.

STEPwise approach to non-communicable diseases risk factor surveillance known as STEPs focus on risk factors for non-communicable diseases in adults more than 18 years old [18]. We used all 6 phases of this survey conducted in years 2005, 2007, 2008, 2009, 2011 and 2016 [19].

In order to cover all target population, we add the information of childhood and adolescence surveillance and prevention of adult non-communicable disease (CASPIAN study). This survey follows the WHO, global school-based student health survey (GHSH) instructions and cover adolescences population at school age [20]. Data for CASPIAN-III (2009–2010), CASPIAN-IV (2011–2012) and CASPIAN-V (2015) were used [21].

Finally, we entered the urbanization proportion to model as the proxy indicator for differentiation between urban–rural lifestyle. This variable derived from population dataset which is estimated based on census information. We defined it as the ratio of population living in urban areas to population living in rural areas. All the data sources are nationally representative surveys that were based on international health organization guidelines.

### Covariates

We extracted a list of potential covariates that could cooperate in modeling. In the first step, we calculated all the covariate values at the individual level, then aggregated them to construct a data set for all the combinations of the province, year, age, and sex. In case of unavailable real data, we estimated the values using a nonparametric smoothing approach, spline. In this manner, we used the spline function in R statistical software and computes a monotone cubic spline using Hyman filtering [22]. The smoothing and estimation of covariates were conducted in all levels of province-age-sex combinations across the year.

The BMI was computed as weight in kilograms divided by the square of height in meters. The smoking history is defined as if a person smoked any tobacco products during her/his life. The current smoking status is also defined similarly but at the study time. We extracted the key components of food frequency questionnaires, include the appropriate percentage of using fruit, vegetables, and fish. In this part, we used the prevalence of less than five total servings (400 g) of fruit and vegetables per day and non-weekly fish consumption as the risk factors for non-communicable diseases.

Blood pressure measurements enter directly into the models as the means of systolic and diastolic blood pressure. In addition, the prevalence of high blood pressure in the sub-populations was added to the covariates list. The same approach was considered for entering fasting blood glucose. Both glucose level and prevalence of type 2 diabetes mellitus were made for modeling.

### Statistical modeling

We used a mixed-effects Poisson regression in order to model the data and estimate the incidence rates [23]. The separate models were fitted for each type of malignant neoplasms. The number of new cases were modeled against the fixed effect of covariates. In addition, the fixed effects of age groups entered the model as dummy variables. The correlation between incident cases across times and unknown causes of variations within the provinces were captured by the random effect of year and provinces respectively. Finally, the population at risk entered as the offset in the model.

### Model building and validation

A backward elimination approach was used to select the best subset of covariates that should remain in the model. In order to select the best format of entering fasting blood glucose and blood pressure, we fitted 4 different starting full models and then reduced these models to find the best one. These 4 models considered all other covariates in addition to 1) mean of Fasting Blood Glucose (FBG), Systolic blood pressure (SBP), and Diastolic Blood Pressure (DBP) or 2) mean of FBG, the prevalence of hypertension, or 3) mean of SBP and DBP and the prevalence of diabetes or 4) the prevalence of diabetes and hypertension. In this way, we prevented entering collinear variables into the model. Models were compared using Akaike information criterion (AIC) and Bayesian information criterion (BIC) criteria.

The model prediction power and validity were explored using a fivefold cross-validation approach. At first, the dataset was divided randomly into 5 subsets. Then at each step, four-part of these subsets were used to model building and the other one for checking the results. The root means the squared error was used to evaluate the models. We used a similar approach to select the best definition of age groups.

### Ethical consideration

This study was authorized by the ethical committee of Tehran University of Medical Sciences (IR.TUMS.VCR.REC.1398.218). Recruited participants’ data is protected by all authors. No individual data is reported since results are created using statistical modeling. Participants also provided informed consents.

## Results

### Malignant neoplasms of female genital organs

Cervix uteri has the highest age-specific incidence rate in 2005 with 12.22 (10.9–13.54) in the 65–74 years old age group per 100,000 females. Corpus uteri has the highest age-specific incidence rates in 2010, 13.88 (13.5–14.27) and 2015, 15.98 (15.4–16.55) for age groups 55–64 and 65–74 years old respectively. Ovarian neoplasm in the age group 65–74 years with the rate of 20.57 (19.94–21.19) has the highest incidence rate in 2020.

Supplementary Fig. 1 depicts the changing trend of age-specific incidence rates across all years for all types of female’s genital malignant neoplasm. The crossing lines is a sign of a changing age pattern of incidence rates across years. For instance, the incidence rate of malignant neoplasm of the vulva was higher in 75 + until 2018 and after this time in 65–74 years. Albeit, the distance between these two groups is going to decrease over time.

**Fig. 1:**
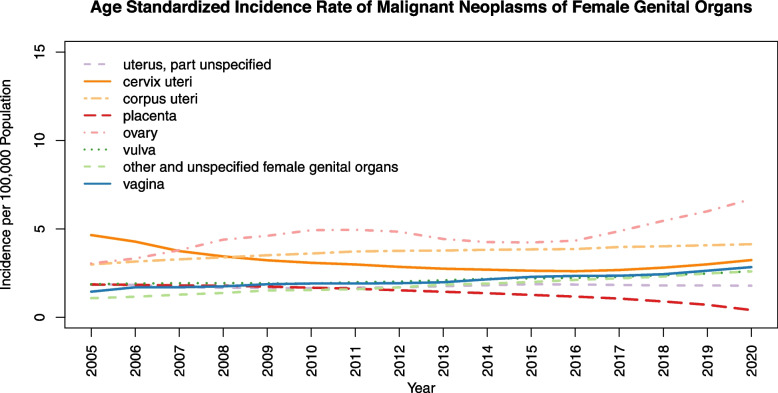
Age Standardized Incidence Rate of Malignant Neoplasms of Female Genital Organs in 100,000 female population

The neoplasm of cervix uteri shows the highest age-standardized incidence rate of 4.65 (4.23–5.10) in 2005. The incidence of this neoplasm is almost declining over the years (Fig. 1). Such that it becomes the third most occurring neoplasm with an incidence rate of 3.24 (2.99–3.51) in 2020 after ovarian and corpus uteri neoplasm with 6.69 (6.32–7.06) and 4.14 (3.86–4.43) respectively. In addition, vagina neoplasm slightly increases, and placenta neoplasm decreases over the years. The age-standardized incidence rate of vulva and unspecified part of the uterus neoplasms are almost constant.

**Fig. 2:**
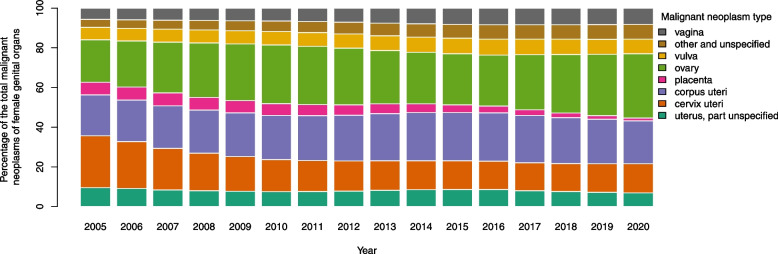
Percentage of each type of malignant neoplasms from the total female genital organ malignant neoplasms

Percentages of share for each type of neoplasms from the total female genital organs neoplasms across years are presented in Fig. 2 as a stacked bar plot. The largest and the smallest share of malignant neoplasms in 2005 belong to cervix uteri and other unspecified neoplasms with 26.19% and 4.06% respectively. Ovarian neoplasm share increase from 21.37% corresponding to ranked 2 in 2005 to 32.94% corresponding to the first rank in 2020. On the other hand, corpus uteri placed in the second rank of female genital neoplasm in 2020 with 21.61% of total incidence cases.

**Fig. 3:**
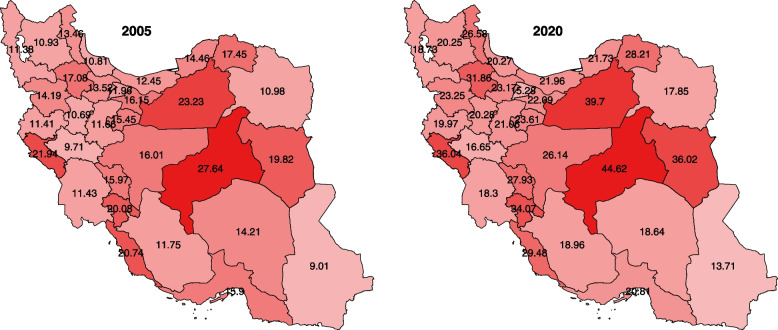
Geographical distribution of female genital organs neoplasms incidence rates in 2005 and 2020

Geographical distribution of incidence rates across provinces in 2005 and 2020 for female genital neoplasms of Iran are available in Fig. 3. All provinces show the increasing trend of incidence rate.

Supplementary Fig. 2 shows the age-specific and all ages incidence rates of four male genital malignant neoplasms per 100,000 males for 2005, 2010, 2015, and 2020. The malignant neoplasm of the prostate has the highest incidence rate in the ages after 45 years, while testis neoplasm was responsible for the most incident cases in the earlier age groups. The incidence rate of the prostate, testis, and penis neoplasms increased over time. The estimated incidence rate of prostate neoplasm is 12.15 (11.97–12.34) in 2005 and 31.36 (31.23–31.53) in 2020.

**Fig. 4:**
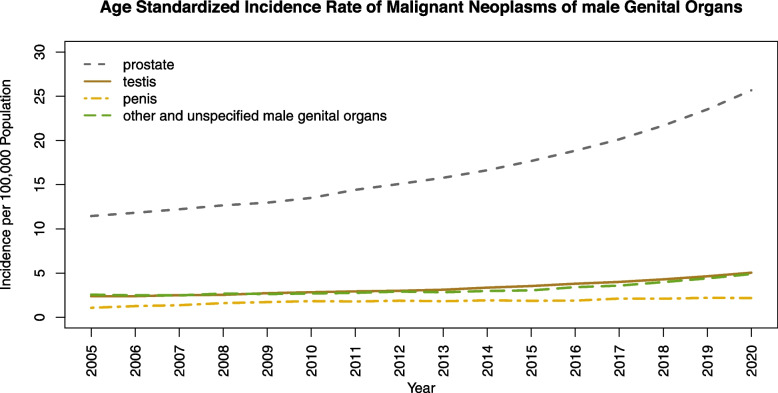
Age Standardized Incidence Rate of Malignant Neoplasms of Male Genital Organs in 100,000 male population

Age-standardized time trends of incidence rate per 100,000 male population are depicted in Fig. 4. The highest values and the most increasing rate are related to the prostate neoplasm that increases from 11.46 (10.87–12.07) in 2005 to 25.67 (24.96–26.40) in 2020. The age-standardized incidence rate of penis neoplasm shows the least values in the years of study. The incidence rate in 2020 is 2.19 (1.94–2.45) that is twice the incidence rate in 2005 with a value of 1.08 (0.87–1.32).

**Fig. 5:**
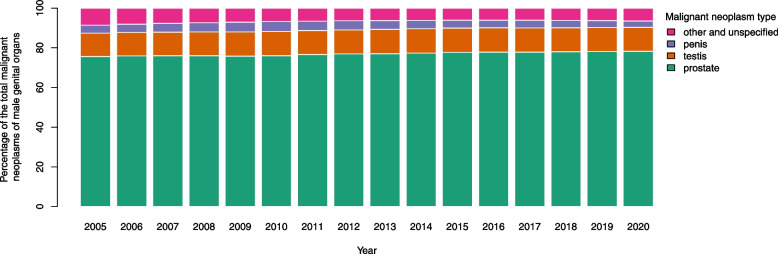
Percentage for each type of malignant neoplasms from the total male genital organs malignant neoplasms

The proportions from the total malignant neoplasms of male genital organs are depicted in Fig. 5. This proportion is almost constant over the years. It varies from 75.56% in 2005 to 78.24% in 2020 for prostate neoplasm. The highest proportion of testis neoplasm is related to 2014 with 12.29% and the lowest one is 11.83% for 2005. The penis neoplasm reaches the highest and the lowest proportion in 2010 and 2020 with 4.99% and 3.29% respectively.

**Fig. 6:**
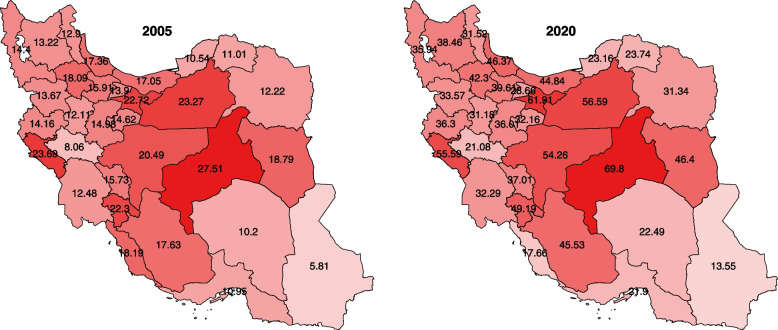
Geographical distribution of male genital organs neoplasms incidence rates in 2005 and 2020

Finally, the geographical distribution of male genital organs neoplasms incidence rates for the first and last year of the study is depicted in Fig. 6.

## Discussion

In this study, we conducted an overview of national and subnational incidence rate combined with trends for each type of gynecological cancers in both men and women from 2005 to 2020 in Iran. The total number of cancer incidences in both men and women has increased over the past 15 years. There is a rising trend in the incidence rate of ovarian and vagina cancer as well as corpus uteri while the incidence rate for cervix uteri has decreased over the years. Our results indicate an increase in the incidence of all male cancer types but most notably prostate and testis cancer.

In our study in cancers related to women, Cervix uteri showed a decreasing trend from 2005 to 2017 with a mild increase from then to 2020. It has fall from the first to the third place of cancers with the most numbers of incidence in woman behind Ovary cancer and Corpus Uteri, which can be explained with expanding compliance with pap smear screening test and the decreased age for starting screening in women resulting in early diagnosis and detection of pre-malignant lesions [24]. When comparing our data to global findings, the incidence rate for cervical cancer is decreasing over the past decade but it still has the highest numbers of new cases annually [2, 25]. As for the Ovarian cancer, there is an increasing trend from 2005 to 2011 followed with a mild decrease in incidence until 2016 and a sudden increase afterwards. Thus, making it the most common genital cancer in Iranian women. This is in line with other studies performed in Iran [7, 26, 27]. The incidence for ovarian cancer has increased globally while Asia is accountable for more than half the incidences reported (51.8%) followed by Europe (22.9%) [28, 29]. Obesity is a well-known risk factor for ovarian cancer [30, 31]. There is also a strong correlation between Human Development Index (HDI) and life expectancy index with incidence for ovarian cancer [28], while increase in HDI is resulted in reduced number of incidences for cervical cancer [32]. Ovarian cancer is usually detected too late and at advanced stages, since there is no specific symptoms at early stages or a sensitive screening test [33]. Despite the recent decline in cervical cancer trend in Iran, it is still responsible for 21% of all women genital cancers and is the most common in the world. The highest incidence and mortality are in Africa and incidence rates are 7 to 10 times lower in Northern America, Australia, and Western Asia [2, 34]. Patient awareness and easy and affordable screening for cervical cancer using pap smear tests, have helped a lot with early diagnosis and proper treatment of the disease, resulting in decreased mortality rate of cervical cancer around the world [24]. Unlike developed countries, HPV (Human papillomavirus) vaccination at population level is not an optimal choice for cervical cancer prevention in Iran as it is not cost-effective [35–37].

As for the male genital cancers, prostate cancer has the highest numbers of incidence, being responsible for more than 75% of all male genital cancers followed by testis cancer (12%). Although according to age-specific pattern, prostate cancer is the most common type of cancer in 45 years and more while in earlier group ages, testis cancer is more common. Comparing our results to global findings, the incidence rate of prostate cancer varies from 6.3 to 83.4 per 100,000 men across regions. The highest rates found in Western Europe, and Northern America countries and the lowest rates in Asia and Northern Africa. Elderly, family history of this cancer, lifestyle factors such as smoking, obesity, nutritional status may increase the risk of advanced prostate cancer [2, 38]. This might be due to differences in usage of Prostate-Specific Antigen (PSA) testing in each region [39]. Genetic factors are pivotal in occurrence of prostate cancer [40]. Moreover, Chu et al. [41] suggests the incidence for prostate cancer in African-Americans to be 40 times higher comparing with African men. Aside from the possibilities of underdiagnosis or lack of proper healthcare system and valid registries, this indicates the importance of environmental factor along with genetics. Westernized life style and physical inactivity have a positive correlation with prostate cancer incidence [42]. There are some suggestions regarding lifestyle or dietary changes [43, 44], but there are no proven prevention methods for prostate cancer and PSA serum marker is currently the best clinical monitoring method for early diagnosis.

The strength of this study is the duration of data collection, demonstrating the incidence trend for each individual neoplasm for both age-standardized rate and age-specific rate groups. In addition, we had access to individual data from the national cancer registry. It facilitated our data process and modeling part. We ensured that the data had the most possible completeness, fewer missing values, and that all the relevant fields were gathered. Also gathering data for each province separately, has helped to identify regions with most incidents. This will benefit health care system and policy makers as to where to use resources that is most needed. One limitation we faced was the incomplete or missing data in our registry. Statistical models were used to extrapolate the missing data. The proportion of missing data is not high and is within acceptable range comparing with similar studies in Iran and worldwide [7, 45, 46]. Another limitation of our work was the inability to specify all malignant neoplasms and cancer types and therefore reporting a portion of malignant neoplasm as “malignant neoplasm of unspecified male /female genital organs” although this was the case for a small portion of samples.

## Conclusions

We conducted a study to observe the changing trend for each genital malignant neoplasm for a duration of 15 years in both men and women. Our study contained data for each age subgroups as well as trend for cancer incidence in every province of Iran over 15 years. Prostate cancer and Ovarian cancer were the most common cancer in 2020 in men and women respectively. Considering Iran as an aging population, the incidence rate is estimated to increase over next decades. Patient awareness and early screening are essential in reducing mortality and expenses forced upon patients and health care system.

## Supplementary Information

**Additional file 1: Supplementary Figure 1.** Age Specific Trends of Incidence Rate of Malignant Neoplasms of Female Genital Organs in 100,000 female population.

**Additional file 2: Supplementary Figure 2.** Age Specific Trends of Incidence Rate of Malignant Neoplasms of male Genital Organs in 100,000 male population.

**Additional file 3: Supplementary Figure 3.** Geographical guide of Iranian provinces.

## Data Availability

We would like to inform all the reviewers that our data, analytic methods, and study materials are available upon request, by contacting our corresponding author.

## Abbreviations

STEPS: STEPwise approach to non-communicable diseases risk factor surveillance
CASPIAN: Childhood and adolescence surveillance and prevention of adult non-communicable disease
GBD: Global burden of diseases
SCI: Statistical Center of Iran
WHO: World Health Organization
GHSH: Global school-based student health survey
FBG: Fasting Blood Glucose
SBP: Systolic blood pressure
DBP: Diastolic Blood Pressure
AIC: Akaike information criterion
BIC: Bayesian information criterion
HDI: Human Development Index
HPV: Human papillomavirus
PSA: Prostate-Specific Antigen

## References

[1] Bray F, Laversanne M, Weiderpass E, Soerjomataram I (2021). The ever-increasing importance of cancer as a leading cause of premature death worldwide. Cancer.

[2] Sung H, Ferlay J, Siegel RL, Laversanne M, Soerjomataram I, Jemal A, Bray F (2021). Global cancer statistics 2020: GLOBOCAN estimates of incidence and mortality worldwide for 36 cancers in 185 countries. CA Cancer J Clin.

[3] Kocarnik JM, Compton K, Dean FE, Fu W, Gaw BL, Harvey JD, Henrikson HJ, Lu D, Pennini A, Xu R (2022). Cancer incidence, mortality, years of life lost, years lived with disability, and disability-adjusted life years for 29 cancer groups from 2010 to 2019: a systematic analysis for the global burden of disease study 2019. JAMA Oncol.

[4] Farhood B, Geraily G, Alizadeh A (2018). Incidence and mortality of various cancers in Iran and compare to other countries: a review article. Iran J Public Health.

[5] Siegel RL, Miller KD, Fuchs HE, Jemal A (2021). Cancer statistics, 2021. CA Cancer J Clin.

[6] Arbyn M, Weiderpass E, Bruni L, de Sanjose S, Saraiya M, Ferlay J, Bray F (2020). Estimates of incidence and mortality of cervical cancer in 2018: a worldwide analysis. Lancet Glob Health.

[7] Eftekharzadeh S, Ebrahimi N, Samaei M, Mohebi F, Mohajer B, Sheidaei A, Gohari K, SaeediMoghaddam S, Ahmadi N, MohammadiFateh S (2020). National and subnational trends of incidence and mortality of female genital cancers in Iran; 1990–2016. Arch Iran Med.

[8] Roshandel G, Ghanbari-Motlagh A, Partovipour E, Salavati F, Hasanpour-Heidari S, Mohammadi G, Khoshaabi M, Sadjadi A, Davanlou M, Tavangar SM (2019). Cancer incidence in Iran in 2014: results of the Iranian National Population-based Cancer Registry. Cancer Epidemiol.

[9] Stangelberger A, Waldert M, Djavan B (2008). Prostate cancer in elderly men. Rev Urol.

[10] Ferlay J, Soerjomataram I, Dikshit R, Eser S, Mathers C, Rebelo M, Parkin DM, Forman D, Bray F (2015). Cancer incidence and mortality worldwide: sources, methods and major patterns in GLOBOCAN 2012. Int J Cancer.

[11] Özdemir BC, Dotto GP (2017). Racial differences in cancer susceptibility and survival: more than the color of the skin?. Trends Cancer.

[12] Katzke VA, Kaaks R, Kühn T (2015). Lifestyle and cancer risk. Cancer J.

[13] Kamangar F, Dores GM, Anderson WF (2006). Patterns of cancer incidence, mortality, and prevalence across five continents: defining priorities to reduce cancer disparities in different geographic regions of the world. J Clin Oncol.

[14] Zhang Z (2016). Multiple imputation for time series data with Amelia package. Ann Transl Med.

[15] Hastie T, Tibshirani R, Friedman JH. The elements of statistical learning: data mining, inference, and prediction. 2nd Ed. Stanford, California: Springer; 2009.

[16] Census 2016 - general results [website]. Statistical Center of Iran; 2016. https://www.amar.org.ir/english/Population-and-Housing-Censuses/Census-2016-General-Results. Accessed 7 Mar 2023.

[17] Shyu E, Caswell H (2014). Calculating second derivatives of population growth rates for ecology and evolution. Methods Ecol Evol.

[18] World Health Organization, Noncommunicable D, Mental Health C (2005). WHO STEPS surveillance manual: the WHO STEPwise approach to chronic disease risk factor surveillance / Noncommunicable Diseases and Mental Health, World Health Organization.

[19] Djalalinia S, Modirian M, Sheidaei A, Yoosefi M, Zokaiee H, Damirchilu B, Mahmoudi Z, Mahmoudi N, Hajipour MJ, Peykari N (2017). Protocol design for large-scale cross-sectional studies of surveillance of risk factors of non-communicable diseases in Iran: STEPs 2016. Arch Iran Med.

[20] Beal T, Morris SS, Tumilowicz A (2019). Global patterns of adolescent fruit, vegetable, carbonated soft drink, and fast-food consumption: a meta-analysis of global school-based student health surveys. Food Nutr Bull.

[21] Kelishadi R, Heshmat R, Farzadfar F, EsmaeilMotlag M, Bahreynian M, Safiri S, Ardalan G, RezaeiDarzi E, Asayesh H, Rezaei F (2017). Prevalence of cardio-metabolic risk factors in a nationally representative sample of Iranian adolescents: the CASPIAN-III study. J Cardiovasc Thorac Res.

[22] Smith L, Hyndman RJ, Wood SN (2004). Spline interpolation for demographic variables: the monotonicity problem. J Popul Res.

[23] Agresti A. Analysis of ordinal categorical data. 2nd Ed. Wiley; 2010. Online ISBN:9780470594001. 10.1002/9780470594001.

[24] Yang DX, Soulos PR, Davis B, Gross CP, Yu JB (2018). Impact of widespread cervical cancer screening: number of cancers prevented and changes in race-specific incidence. Am J Clin Oncol.

[25] Lin S, Gao K, Gu S, You L, Qian S, Tang M, Wang J, Chen K, Jin M (2021). Worldwide trends in cervical cancer incidence and mortality, with predictions for the next 15 years. Cancer.

[26] Sharifian A, Pourhoseingholi MA, Norouzinia M, Vahedi M (2014). Ovarian cancer in Iranian women, a trend analysis of mortality and incidence. Asian Pac J Cancer Prev.

[27] Moradi Y, Jafari M, Chaichian S, Khateri S, Akbarian A, Moazzami B, Mansori K, Mahmodi Y, Samie S (2016). Trends in ovarian cancer incidence in Iran. Int J Cancer Manag.

[28] Khazaei Z, Namayandeh SM, Beiranvand R, Naemi H, Bechashk SM, Goodarzi E (2021). Worldwide incidence and mortality of ovarian cancer and Human Development Index (HDI): GLOBOCAN sources and methods 2018. J Prev Med Hyg.

[29] Zhang Y, Luo G, Li M, Guo P, Xiao Y, Ji H, Hao Y (2019). Global patterns and trends in ovarian cancer incidence: age, period and birth cohort analysis. BMC Cancer.

[30] Olsen CM, Green AC, Whiteman DC, Sadeghi S, Kolahdooz F, Webb PM (2007). Obesity and the risk of epithelial ovarian cancer: a systematic review and meta-analysis. Eur J Cancer.

[31] Protani MM, Nagle CM, Webb PM (2012). Obesity and ovarian cancer survival: a systematic review and meta-analysis. Cancer Prev Res.

[32] Singh GK, Azuine RE, Siahpush M (2012). Global inequalities in cervical cancer incidence and mortality are linked to deprivation, low socioeconomic status, and human development. Int J MCH AIDS.

[33] Patni R (2019). Screening for ovarian cancer: an update. J Midlife Health.

[34] Hwang JY, Lim WY, Tan CS, Lim SL, Chia J, Chow KY, Chay WY (2019). Ovarian cancer incidence in the multi-ethnic Asian city-state of Singapore 1968–2012. Asian Pac J Cancer Prev.

[35] Khatibi M, Rasekh HR, Shahverdi Z, Jamshidi HR (2014). Cost-effectiveness evaluation of quadrivalent human papilloma virus vaccine for HPV-related disease in Iran. Iran J Pharm Res.

[36] Majidi A, Ghiasvand R, Hadji M, Nahvijou A, Mousavi A-S, Pakgohar M, Khodakarami N, Abedini M, AmouzegarHashemi F, RahnamayeFarzami M (2015). Priority setting for improvement of cervical cancer prevention in Iran. Int J Health Policy Manag.

[37] Yaghoubi M, Nojomi M, Vaezi A, Erfani V, Mahmoudi S, Ezoji K, Zahraei SM, Chaudhri I, Moradi-Lakeh M (2018). Cost-effectiveness analysis of the introduction of HPV vaccination of 9-year-old-girls in Iran. Value Health Reg Issues.

[38] Ha Chung B, Horie S, Chiong E (2019). The incidence, mortality, and risk factors of prostate cancer in Asian men. Prostate Int.

[39] Quinn M, Babb P (2002). Patterns and trends in prostate cancer incidence, survival, prevalence and mortality. Part I: international comparisons. BJU Int.

[40] Langeberg WJ, Isaacs WB, Stanford JL (2007). Genetic etiology of hereditary prostate cancer. Front Biosci.

[41] Chu LW, Ritchey J, Devesa SS, Quraishi SM, Zhang H, Hsing AW (2011). Prostate cancer incidence rates in Africa. Prostate Cancer.

[42] Baade PD, Youlden DR, Krnjacki LJ (2009). International epidemiology of prostate cancer: geographical distribution and secular trends. Mol Nutr Food Res.

[43] Mills PK, Beeson WL, Phillips RL, Fraser GE (1989). Cohort study of diet, lifestyle, and prostate cancer in Adventist men. Cancer.

[44] Wilson KM, Giovannucci EL, Mucci LA (2012). Lifestyle and dietary factors in the prevention of lethal prostate cancer. Asian J Androl.

[45] Danaei G, Finucane MM, Lin JK, Singh GM, Paciorek CJ, Cowan MJ, Farzadfar F, Stevens GA, Lim SS, Riley LM (2011). National, regional, and global trends in systolic blood pressure since 1980: systematic analysis of health examination surveys and epidemiological studies with 786 country-years and 5·4 million participants. Lancet.

[46] Finucane MM, Stevens GA, Cowan MJ, Danaei G, Lin JK, Paciorek CJ, Singh GM, Gutierrez HR, Lu Y, Bahalim AN (2011). National, regional, and global trends in body-mass index since 1980: systematic analysis of health examination surveys and epidemiological studies with 960 country-years and 9·1 million participants. Lancet.

