# Supplementary material for: The 15-year national trends of genital cancer incidence among Iranian men and women; 2005–2020
Source: BMC Public Health. 2023 Mar 15;23:495. doi: 10.1186/s12889-023-15417-0 (PMC10015665; doi:10.1186/s12889-023-15417-0)
Supplement: Supplementary file 2 — Additional file 2: Supplementary Figure 2. Age Specific Trends of Incidence Rate of Malignant Neoplasms of male Genital Organs in 100,000 male population. [file 12889_2023_15417_MOESM2_ESM.pdf]

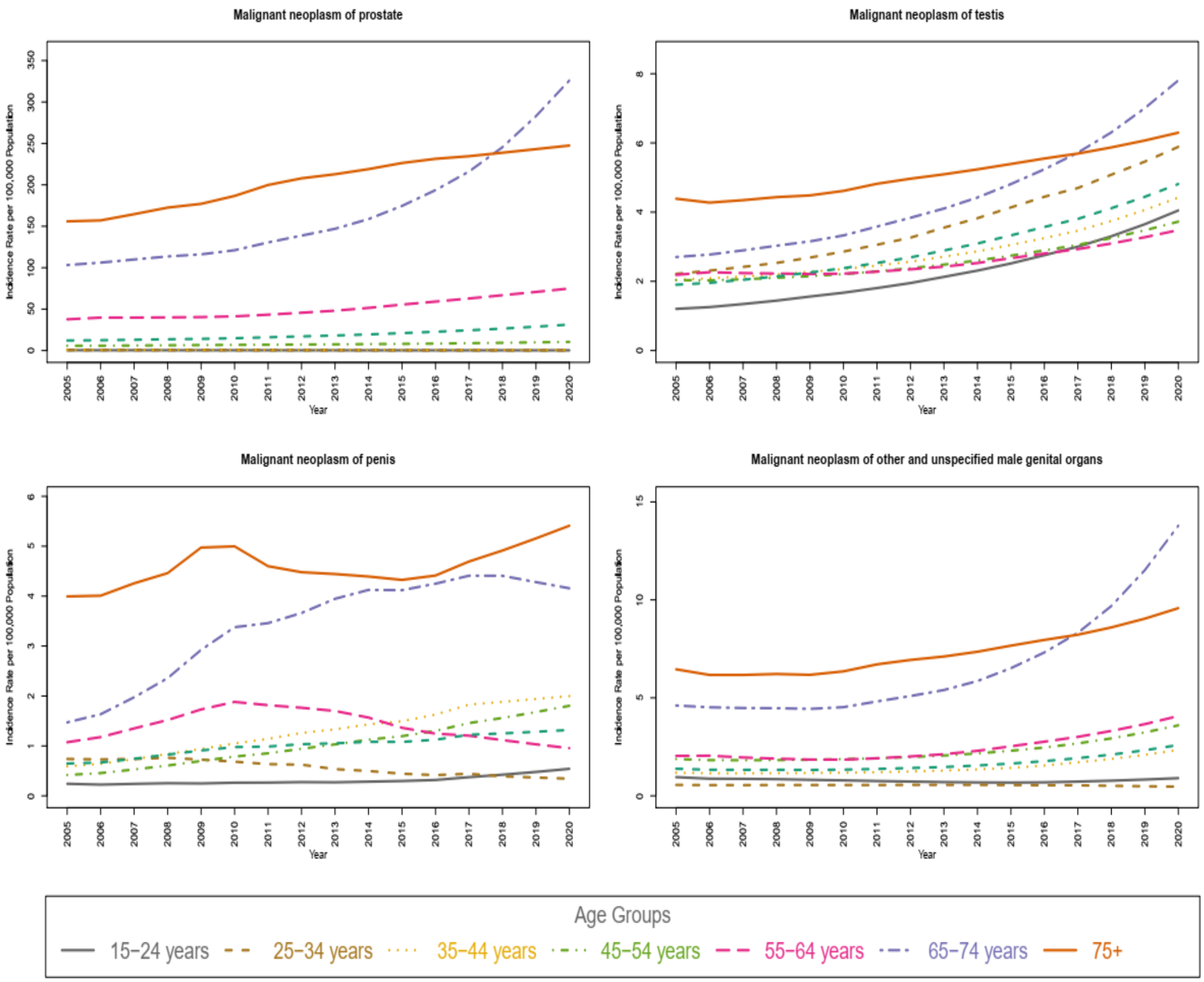

Supplementary figure 2. Age Specific Trends of Incidence Rate of Malignant Neoplasms of male Genital Organs in 100,000 male population
