# Supplementary figures and images for: The 15-year national trends of genital cancer incidence among Iranian men and women; 2005–2020
Source: BMC Public Health. 2023 Mar 15;23:495. doi: 10.1186/s12889-023-15417-0 (PMC10015665; doi:10.1186/s12889-023-15417-0)

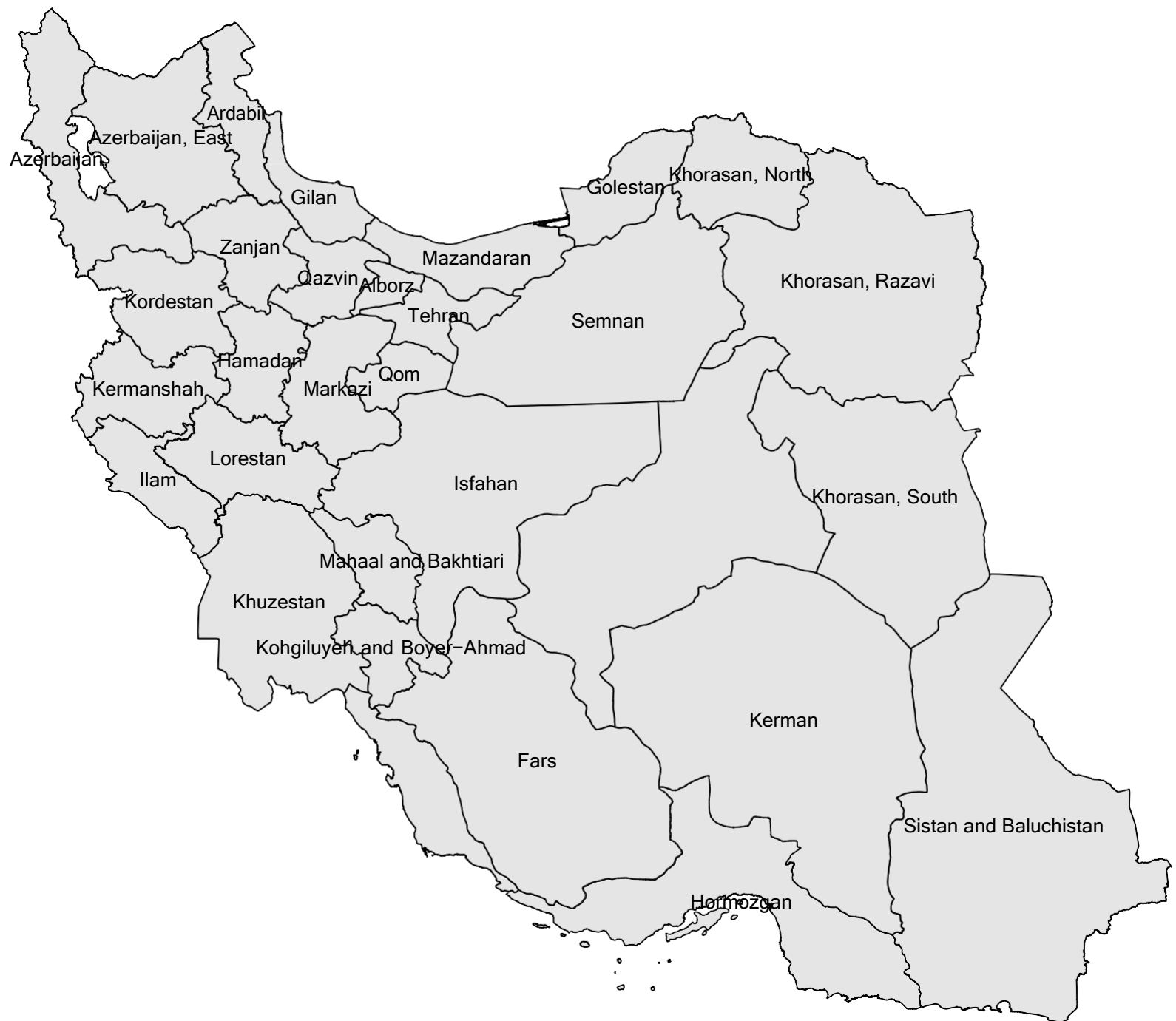

Supplementary figure 3. Geographical guide of Iranian provinces

Supplement: Supplementary file 3 — Additional file 3: Supplementary Figure 3. Geographical guide of Iranian provinces. [file 12889_2023_15417_MOESM3_ESM.pdf]
